# Challenges in public perception: highlights from the United Kingdom-Brazil Dementia Workshop

**DOI:** 10.1590/1980-57642020dn14-030002

**Published:** 2020

**Authors:** Lucas Nogueira de Carvalho Pelegrini, Abigail Hall, Emma Hooper, Déborah Oliveira, Flora Guerra, Francine Golghetto Casemiro, Janine Bonfadini, Keir Yong, Natalie Pereira, Raquel Costa, Maira Tonidandel Barbosa, Eneida Mioshi

**Affiliations:** 1 Universidade de São Paulo - Ribeirão Preto, SP, Brazil.; 2 Exeter University - Exeter, United Kingdom.; 3 University of Manchester - Manchester, United Kingdom.; 4 Universidade Federal de São Paulo - São Paulo, SP, Brazil.; 5 Prefeitura de Belo Horizonte - Belo Horizonte, MG, Brazil.; 6 Universidade Federal do Ceará - Fortaleza, CE, Brazil.; 7 University College London - London, United Kingdom.; 8 Pontifícia Universidade Católica do Rio Grande do Sul - Porto Alegre, RS, Brazil.; 9 Universidade de São Paulo - São Paulo, SP, Brazil.; 10 Universidade Federal de Minas Gerais - Belo Horizonte, MG, Brazil.; 11 University of East Anglia - Norwich, United Kingdom.

**Keywords:** dementia, cognitive impairment, Alzheimer’s disease, aged, social stigma, social perception, demência, comprometimento cognitivo, doença de Alzheimer, idoso, estigma social, percepção social

## Abstract

In July 2019, Belo Horizonte hosted an international workshop for 27 junior researchers, whose participants were from Brazil and the United Kingdom. This three-day meeting organized by the Universidade Federal de Minas Gerais and the University of East Anglia addressed challenges in cognitive impairment and dementia, with particular interest in public perceptions, diagnosis and care management. The purpose of this report is to highlight the outcomes of the above-mentioned workshop regarding the topic of public perceptions (part I). Discussions focused on differences and similarities between countries, as well as on identifying main issues that required collaborative and creative solutions. After these group discussions, four core themes emerged: I) cognitive impairment; II) dementia - beyond Alzheimer’s disease; III) prevention; and IV) stigma. National and international initiatives to deal with public misperceptions about cognitive impairment and dementia were discussed.

## RESUMO.

Em julho de 2019, Belo Horizonte sediou um *workshop* internacional cujos participantes foram 27 pesquisadores do Brasil e do Reino Unido. Essa reunião de três dias, organizada pela Universidade Federal de Minas Gerais e pela Universidade de East Anglia, abordou os desafios no comprometimento cognitivo e na demência, com interesse particular nas *percepções do público, diagnóstico* e *gerenciamento de cuidados*. O objetivo deste relatório foi destacar os resultados do workshop sobre o tópico *percepções do público (parte I)*. O enfoque das discussões foi nas semelhanças e diferenças entre os países, bem como na identificação dos principais problemas que necessitam soluções criativas e colaborativas. Após essas discussões em grupo, quatro temas principais foram elencados: I) comprometimento cognitivo; II) demência - além da doença de Alzheimer; III) prevenção; e IV) estigma. Foram discutidas iniciativas nacionais e internacionais para lidar com as percepções alteradas do público sobre comprometimento cognitivo e demência.

## INTRODUCTION

Dementia has become a global public health priority due to its increasing prevalence and incidence.^1^ ^,^ ^2^ According to the World Health Organization (WHO),^1^ the number of people living with dementia will triple by 2050, with most of these living in low and middle-income countries. National dementia plans which carefully consider societal, demographic and attitudinal factors are needed to inform effective public health responses for dementia.^2^ ^,^ ^3^

Research centres, universities, governments, charity/voluntary health organisations, and patient advocacy groups are trying to provide society with as much information about this topic as possible. Although public awareness about dementia is improving overall, people still have misperceptions about dementia, which may lead to higher underdiagnosis rates, stigma and poor-quality care.^4^ ^,^ ^5^ Middle- and low-income countries will face greater challenges, due to particular limitations in publicity and available information regarding dementia.

Aims from the WHO’s Global Action Plan include developing public policies, national strategies and public awareness campaigns regarding dementia. In this context, considering challenges in public perception and lessons learnt from awareness campaigns in the United Kingdom and in Brazil, as well as considering commonalities and differences between these countries, a subgroup from the workshop including participants from both countries shared experience and discussed future perspectives on this topic during the three-day event. Correspondingly, the purpose of this paper is to provide the highlights of discussions on *public perception* that resulted from this meeting.

## THE UNITED KINGDOM-BRAZIL DEMENTIA WORKSHOP

“Challenges in cognitive impairment and dementia: (mis)perceptions, (mis)diagnosis and care management” was the main theme of the First United Kingdom-Brazil Dementia Workshop. This three-day event took place in Belo Horizonte, Minas Gerais, Brazil, in July 2019. Attendees were 27 junior researchers from the United Kingdom and Brazil with various training backgrounds (nurses, gerontologists, psychologists, occupational therapists, physical therapists, speech and language therapists, geriatricians, neurologists, and psychiatrists) and six faculty members from the Universidade Federal de Minas Gerais (UFMG) and the University of East Anglia (UEA) who acted as mentors. In addition to the creation of an international network group, the purpose of this event was to facilitate the discussion about creative and collaborative solutions to address challenges in diagnosis, public perceptions and dementia care.

This paper reports on the discussions held during this three-day workshop, which involved challenges in public perceptions, with emphasis on the following issues: cognitive impairment: misdiagnosis and overdiagnosis, dementia: beyond Alzheimer’s disease (AD), prevention, stigma, possible solutions. It is hoped that this report can provide researchers, health professionals and public policy makers with ideas to support dementia-related initiatives, both nationally and internationally.

## COGNITIVE IMPAIRMENT

### Under-recognition

One of the key issues regarding public perceptions of cognitive impairment is under-recognition, which is a serious and potentially life-limiting health problem. In Brazil, most people believe that ageing is associated with the presence of illnesses and disabilities, such as cognitive impairment. Consequently, older adults tend to be less likely to seek health services for cognitive deficits alone.^6^ Instead, people with cognitive complaints tend to seek help only when their functioning has become impaired, a tendency which was considered to be less apparent in the UK. In addition, even if such individuals seek the help of general practitioners (GPs), these professionals may have limited familiarity and/or training regarding cognitive impairment and dementia in both the UK and Brazil.^7^ ^,^ ^8^

A Brazilian study about factors influencing delay in the diagnosis of cognitive impairment showed a median delay of 1.5 to 1.8 years for the diagnosis of dementia (AD) at a tertiary ambulatory clinic in a major city.^9^ Miranda and colleagues observed an excessive time between onset and proper diagnosis and, of those who believed there was a delay in diagnosis, about 36% stated that the “family thought the changes were normal for the age of the patient”, while 45.3% reported that the “doctor did not reach a diagnosis”.^9^ Unfortunately, many doctors in primary care do not fully understand the diagnostic criteria for dementias. In addition, few studies have investigated public awareness about dementia worldwide. A study of public awareness of dementia was conducted in Botucatu, São Paulo, Brazil, in which individuals answered a questionnaire about characteristics of healthy elderly and individuals with dementia. Surprisingly, even upon suspecting dementia, only a few participants stated they would seek specialized medical help.^10^ Therefore, lack of knowledge usually leads to stigmatization of patients and lack of family support, as well as inappropriate recognition and management of dementia in health services.^11^

### Misdiagnosis

Misdiagnosis in both the UK and Brazil was largely attributed to the people and also to health professionals’ underappreciation of the heterogeneity in cognitive symptoms and their underlying pathologies.^12^ Attendees reported professionals’ tendency to misinterpret cognitive symptoms as predominantly or solely related to memory dysfunction. For instance, this includes word finding difficulties being interpreted as ‘forgetting words’, visuoperceptual and visuospatial impairment considered to reflect ‘forgetting’ what or where objects are - even when presented in clear view and disinhibited, or other behaviour disturbances being attributed to ‘forgetting’ what is socially appropriate. People commonly use the term “senile dementia” to refer to older people’s memory problem, as they believe this occurs naturally in later life.

Conversely, underappreciation of how cognitive symptoms might arise from other, sometimes reversible causes such as adverse responses to medication, infection, sleep apnoea, liver or kidney failure, depression or stroke, was reported in both the UK and Brazil.^13^ Lack of access to screening visits to rule out other causes of cognitive dysfunction exacerbates this risk, particularly in Brazil. Finally, socioeconomic characteristics, such as educational background, may also play a role in misdiagnosis.^14^ Firstly, people with low education who have low complexity of day-to-day tasks may not notice a decline in their cognitive function as there is no demand for high-level skills.^15^ Secondly, highly educated people may easily mask cognitive decline or may replace skills due to high cognitive reserve, so dementia may be quite moderate at the time of diagnosis.^16^

### Overdiagnosis

Overdiagnosis was noted in Brazil and the UK, but particularly in the latter, which may also lead to overtreatment. According to the National Institute for Health and Care Excellence (NICE) guidelines,3 memantine is indicated for moderate-to-advanced cases of dementia, but has been prescribed to people at time of initial, early diagnosis. Also noted was the use of acetylcholinesterase inhibitors in people with mild cognitive impairment (MCI), with no evidence of beneficial effects; the recommendation³ is for use in mild-to-moderate AD, for instance. Lack of training may also play a role in overdiagnosis:^17^ misinterpretation of biomarkers is an issue, for instance making a clinical diagnosis of AD based on biomarker evidence of amyloid positivity and co-occurring cognitive symptoms, even when the symptoms might have a non-neurodegenerative cause.^17^

## DEMENTIA - BEYOND ALZHEIMER’S DISEASE

### Terminology

One of the main issues that may affect public perceptions regarding dementia is the lack of understanding about its various terminologies. Often, people do not know that ‘dementia’ is an umbrella term for various brain diseases (e.g. frontotemporal dementia, Lewy body diseases, corticobasal syndrome).^6^ There is also a public confusion about the terms AD and dementia and their overlap (e.g., it is common for individuals to say “she/he has Alzheimer’s, but not dementia”). Another issue related to terminology is the negative connotation of the dementia syndrome at a public level. Terms like ‘dementia’ or ‘demented’ can be stigmatizing in both UK and Brazil. Additionally, it is common to hear flippant/dismissive comments from the general public to refer to normal memory loss in Brazil (e.g. some people say “I am demented” or another similar comment to apologise for forgetting something they should have remembered).^17^ ^,^ ^18^ Such behaviour may contribute to disseminating a negative notion about dementia.

### Beyond memory

There is a common public misperception in Brazil and the UK that dementia only leads to memory impairment. This delays diagnoses based on the existence of other symptoms and causes, like in the case of frontotemporal dementia, in which behavioural symptoms prevail, especially in initial phases. Due to this misperception that dementia will only (or at least initially) affect memory, other symptoms such as language, visual or motor dysfunction (for example, arising in primary progressive aphasia, posterior cortical atrophy or corticobasal degeneration) may be dismissed or overlooked. This results in delayed diagnoses being very common for those whose memory is relatively unaffected in early stages in both countries. Finally, in both Brazil and the UK, it seems that health professionals are not exposed enough to cases of people with dementia during their training years in medical school; consequently, they are not sufficiently prepared to detect cognitive and functional deficits arising from the different brain diseases leading to dementia.^19^

## PREVENTION

### Awareness and education

One of the greatest challenges concerning public perceptions about dementia in both the UK and Brazil is that people are often unaware of the existence of modifiable risk factors for dementia.^20^ Individuals may therefore believe that there is nothing they can do to prevent dementia. In addition to the lack of public awareness about dementia-related risk factors, public perceptions about them appear to vary according to people’s educational level, as mentioned in previous sections. This is particularly relevant in Brazil where there is a large proportion of older adults who are illiterate.^21^ We identified that it is critical to increase public awareness of the risk factors for dementia in order to foster proactive personal and societal management of them; at present, there is a tendency toward a reactive approach, where people seek treatment and support only after they experience the onset of symptoms, and when these symptoms are associated with functional decline.^19^

## STIGMA

The International Alzheimer’s Association has recently released a report on this topic with very interesting results from a survey focusing on knowledge and behaviour towards dementia and how stigma is related to this.^22^ It is known that people with dementia often fail to respond depending on the situation because of the lack of skills or opportunity to do so, and this is usually related to stigma.^23^ Thus, stigma is an issue of great importance that demands attention, and was also a topic discussed during the workshop.

### Socioeconomic differences in perception of stigma

We identified potentially differing perceptions relating to the stigma of dementia across different socio-economic groups. This is present in both the UK and Brazil, but is more overtly evident in the Brazilian context. It was observed that people from lower socio-economic groups are potentially more openly accepting of people living with dementia; that a greater informal support network exists for these people; and, within the context of multimorbidity, dementia is viewed as ‘one more thing’ for older people and public health professionals to manage.^24^ By contrast, people from higher socio-economic groups may display greater concern about outward appearance, and therefore more concern about stigma. This potentially places the person with dementia at a greater risk of being ‘hidden away’ from society, particularly if they experience behavioural and psychological symptoms of dementia.^25^

### Impact of stigma on help-seeking

The perceived stigma amongst the public could impact negatively upon people’s willingness to seek diagnosis and support. This is strengthened by a cross-cultural fear of ageing and of diseases. There is a perception that the professionals who are involved in the diagnostic process deal with “crazy people”, and fear of association with this stigmatizing label could lead to avoidance of help-seeking.^26^

## SUGGESTIONS AND SOLUTIONS

After much discussion and considering both Brazilian and UK cultures, the group came up with some possible suggestions and solutions to the aforementioned issues related to dementia. What appears to be the most promising action to tackle all the issues identified is the promotion of knowledge and awareness across different populations and professionals.

First, developing more sensitive cognitive screening batteries for literate and illiterate individuals would be helpful to detect dementia; technology in this sense would be an important ally. Greater attention should be given to primary health-care services, such as including more trained professionals. In Brazil, for example, community health agents, who are professionals with deep knowledge of the community and therefore are key people to strengthen the relationship between community and the health professionals/system, could be strategic to identify people at risk of developing dementia.^27^ ^,^ ^28^ In addition to training community health agents/ professionals to perform screening correctly, rewarding general practitioners who perform a certain number of assessments (e.g. Mini-Mental State Examinations) may potentially solve part of the misdiagnosis problem. This has been successfully done in the UK. Also, availing of current resources, such as the multidimensional screening available in the Brazilian Primary Care Book number 19,^29^ could improve services and benefit both the health system and the community.

Education strategies for professionals should provide more focus on the different types of brain diseases and dementia. Raising awareness for different types of dementia could potentially mitigate the subjective burden experienced by families, as knowing what to expect could help them cope better with dementia. This could begin with simple steps, such as providing people with easy-to-read guidelines (e.g. ten key messages) for caregivers and primary healthcare professionals. We also suggest that specific training be provided for different professionals at all levels of care. We strongly believe that education can be a relevant, easy, cheap and effective way of raising awareness and improving knowledge to reduce fear, prejudice and stigma. Promoting public and patient empowerment and participation (e.g. through involvement in local councils, advocacy groups or research) might be key to reducing the stigma experienced by people living with dementia and by their families.^30^

To reduce stigma, we also advocate implementing activities that increase the empathy and understanding of the general public concerning people with dementia and their personal experience. Dementia should be more often brought into the public domain and people should be encouraged to talk freely about it without fear. Examples of public campaigns which could foster this may include:

- The use of the blue ‘Forget-Me-Not’ flower to symbolise dementia. In the UK, this symbol can be found in many places including shop entrances, public transport, hospitals or as pin badges to indicate a dementia-friendly environment or a person who is supportive of people living with dementia.
- “Dementia friends” training for the public, including experiential learning to increase empathy.
- Using famous people who are either living with dementia or supporting a family member with dementia as public role models.
- Using initiatives such as dementia awareness week, national dementia day, community action days or memory walks as an opportunity to raise awareness, with an underpinning philosophy of aiming for continuous awareness raising (i.e. one-off initiatives were reported as good but insufficient).
- Using intergenerational initiatives including children’s storybooks about dementia (such as ‘Grandpa is a superhero’) and visits to long-term care facilities in order to educate the general public about dementia during the life course.

Currently, such destigmatizing campaigns are implemented more widely in the UK than in Brazil. We advocated taking a health-promoting - rather than a disease-focused - approach in public awareness campaigns for better brain health. In addition, we discussed the importance of promoting and funding studies about dementia, and talked about the issues regarding unmet health, social, and government needs.^31^

We also discussed the role of the media and entertainment industries in destigmatizing dementia, and the impact of the messages they portray. We highlighted the need for sensitive, but not over-sensationalist messages, and discussed the positive impact that some TV programmes can have, such as the recent ‘Our Dementia Choir’ programme in the UK. We highlighted that there may be great advantages in using forms of media that are accessible across countries, such as social media and YouTube, or soap operas in Brazil, in order to raise awareness about dementia. We advocated that dementia awareness be a core curriculum subject in all health and care training, and that people with dementia be involved in the development of such training.

Dementia and cognitive impairment are topics for present and future consideration. It is clear that much progress needs to be made worldwide to guarantee people’s awareness and to make reliable information available. Also, organizations around the world (e.g. charities, non-governmental organizations, and patients’ advocacy groups) are trying to disseminate knowledge on these topics in order to reduce stigma and improve public perceptions.

During the three-day workshop, in-depth discussions between an international multi-professional and multi-cultural group took place. This paper aimed to summarise and disseminate not only the topics that have been discussed, but also the possible solutions to deal with current issues that both the UK and Brazil have experienced. We hope that this summary can provide information to support national and international initiatives for local authorities, charities and other sectors to overcome challenges in public perceptions.
